# A Case Report of Cytarabine-Induced Red Ear Syndrome

**DOI:** 10.7759/cureus.48707

**Published:** 2023-11-12

**Authors:** Monica Botros, Alan De La Rosa, Sahithi Nadella, Aymara Y Chang, Satish S Maharaj

**Affiliations:** 1 Internal Medicine, Texas Tech University Health Sciences Center El Paso, El Paso, USA; 2 Hematology and Oncology, Texas Tech University Health Sciences Center El Paso, El Paso, USA

**Keywords:** red ear syndrome, ara-c ears, acral erythema, auricular erythema, cytarabine

## Abstract

Cytarabine is an antimetabolite used in the treatment of acute myeloid leukemia which acts by inhibiting DNA synthesis and subsequently cell division. It works on rapidly dividing cells, for that reason, it affects cancer cells, bone marrow and skin cells. Cytarabine has variable cutaneous side effects, the most common one is palmar-plantar erythema which usually presents with a tingling sensation around 5-7 days after cytarabine initiation, followed by erythema and tenderness. Auricular erythema is a rare subtype involving bilateral ears which often presents as ear redness and tenderness as described in the presented case. It is unclear if the skin side effects are related to cytarabine dose or plasma concentration. Most cases of auricular erythema have a benign course and resolve spontaneously. Treatment is mainly conservative. Steroids and antihistamines can be used to speed up recovery given that the pathophysiology is thought to be immediate or due to a delayed hypersensitivity reaction to cytarabine.

## Introduction

Cytarabine, also known as arabinosylcytosine (Ara-C), is a pyrimidine analog used to treat hematological malignancies, particularly acute myeloid leukemia (AML), acute lymphocytic leukemia (ALL), chronic myelogenous leukemia (CML), and non-Hodgkin’s lymphoma. It is considered an anti-metabolite, with various side effects including bone marrow suppression, mucositis, nausea, vomiting, hair loss, and less commonly cutaneous manifestations such as acral erythema. In the present report, we present a case of cytarabine-induced auricular erythema which is considered a rare subtype of acral erythema [1].

## Case presentation

**Figure 1:**
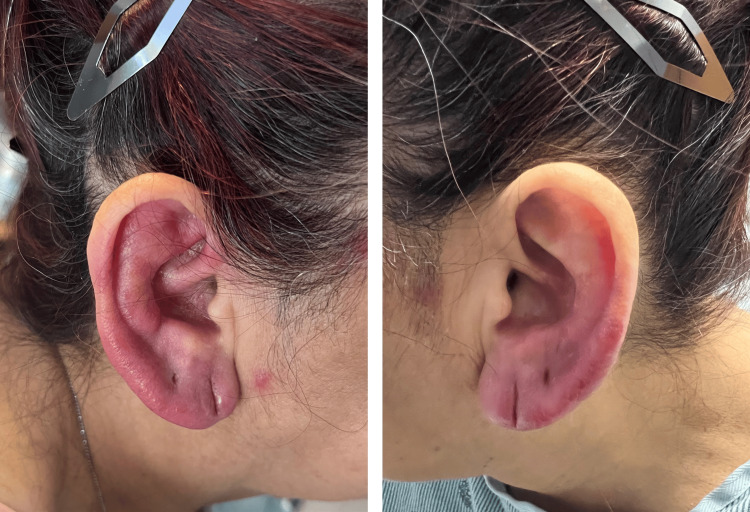
Bilateral red ears prior to oral steroid treatment

**Figure 2:**
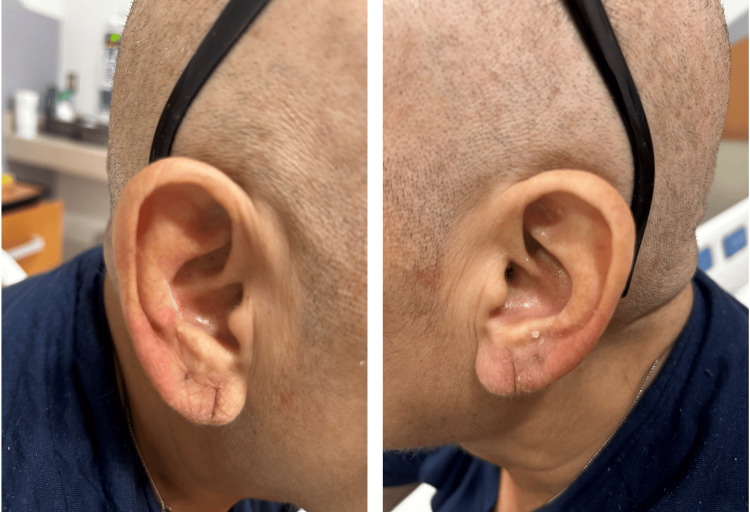
Bilateral ears after seven days of oral steroid treatment with complete resolution of redness

A 63-year-old female with no significant past medical history, presented to the Emergency Department with a complaint of fatigue and recurrent epistaxis for two months. Upon admission, the patient had a white blood count of 70.74 X 103/UL, with 18% myeloblast, anemia and thrombocytopenia. Peripheral smear showed a significant number of blasts with monocytic differentiation, concerning AML. Bone marrow biopsy revealed a hypercellular marrow demonstrating AML with monocytic morphology. The oncology team was consulted, and the patient was started on induction chemotherapy in the form of cytarabine 100mg/m^2^ daily for days 1-7 and idarubicin 12 mg/m^2^ daily for days 1-3. In addition, the patient was started on prophylactic levofloxacin, acyclovir and posaconazole, along with supportive treatment. Two days after completing induction chemotherapy, the patient started complaining of bilateral external ear pain, associated with redness; however, denied any fever or chills. Physical examination was significant for bilateral ear tenderness and redness (Figure 1), which was worsened the following day. A dermatologic examination showed multiple red papules on the forehead, arms and legs. Labs were significant for pancytopenia with absolute neutropenia but with normal kidney and liver functions. Cytarabine-induced auricular erythema versus polychondritis was suspected and the patient was started on cetirizine 10 mg, and prednisone 20 mg for a total of seven days with marked clinical improvement (Figure 2).

## Discussion

Cytarabine is an antimetabolite commonly used alone or in combination with other antineoplastic drugs to treat hematological malignancies, especially AML. It is crucial to identify its side effects for prompt diagnosis and management. In addition to hematological toxicity, cytarabine can cause toxicity to the central nervous system, liver, and skin. The dermatological side effects can present as an immediate or more commonly as a delayed hypersensitivity reaction secondary to release of cytokines [2]. Skin lesions can be in the form of macules, papules or erythema. It can also be diffuse, acral or at flexural level [3]. Cutaneous side effects can be as simple as palmoplantar rash, also known as palmar-planter erythrodysesthesia, or as serious as Stevens-Johnson syndrome, pyoderma gangrenosum or Sweet syndrome [4].

Acral erythema is often self-limiting and responds to symptomatic treatment. On the other hand, serious skin side effects may require high doses of steroids or intravenous immunoglobulin. It is worth mentioning that it is unclear if the skin toxicity is dose-dependent or not, since it was reported previously in patients with low dose Ara-C [1]. There are studies reporting that skin injury is related to both cumulative dose and peak plasma concentration of the chemotherapeutic agent which has a direct cytotoxic effect on the epidermis [5].

The presentation of auricular erythema is usually called Ara-C ears or red ear syndrome (RES) which is a rare subtype of acral erythema. Ara-C ears often happen after the first exposure to the medication, and it gets cleared spontaneously without recurrence upon re-challenge [1]. Treatment is mainly supportive such as pyridoxine and cold compresses, along with topical or oral corticosteroids and antihistamines [5]. Patients' reassurance of the condition’s benign nature will prevent unnecessary intervention and chemotherapy cessation [6].

## Conclusions

Cytarabine-associated RES is a form of palmoplantar-erythrodysesthesia, which is a rare Ara-C-related skin reaction likely secondary to a delayed hypersensitivity reaction. It is important to exclude other differential diagnoses including infectious causes and serious skin reactions when you are suspecting RES. It is a benign self-limiting condition and treatment is mainly conservative with supportive treatment. Patients usually recover spontaneously with no permanent sequelae.
